# Primary prevention of overweight in children and adolescents: a meta-analysis of the effectiveness of interventions aiming to decrease sedentary behaviour

**DOI:** 10.1186/1479-5868-9-61

**Published:** 2012-05-28

**Authors:** Amy van Grieken, Nicole PM Ezendam, Winifred D Paulis, Johannes C van der Wouden, Hein Raat

**Affiliations:** 1 Department of Public Health, Erasmus MC, University Medical Center, P.O. Box 2040, 3000 CA, Rotterdam, The Netherlands; 2 Department of General Practice, Erasmus MC, University Medical Center, P.O. Box 2040, 3000 CA, Rotterdam, The Netherlands

**Keywords:** Sedentary behaviour, Intervention, Overweight prevention, Children, General population

## Abstract

The objectives of this meta-analysis were to provide an overview of the evidence regarding the effects of interventions, implemented in the school- and general population setting, aiming to prevent excessive sedentary behaviour in children and adolescents on (1) the amount of sedentary behaviour and (2) BMI. Differences in effects on sedentary behaviour and BMI between single health behaviour interventions (sedentary behaviour only) and multiple health behaviour interventions were explored.

A literature search was conducted in PubMed, EMBASE, Web of Science, PsycINFO and Cochrane Database of Systematic Reviews. Thirty-four (R)CT studies evaluating 33 general population interventions, published between 1990 and April 2011, aiming to decrease sedentary behaviour in normal weight children or adolescents (0–18 years) were included. Intervention duration ranged from 7 days to 4 years. Mean change in sedentary behaviour and BMI from baseline to post-intervention was calculated using a random effects model.

Results showed significant decreases for the amount of sedentary behaviour and BMI. For sedentary behaviour the post-intervention mean difference was −17.95 min/day (95%CI:-26.61;–9.28); the change-from-baseline mean difference was −20.44 min/day (95%CI:-30.69;–10.20). For BMI the post-intervention mean difference was −0.25 kg/m² (95%CI:-0.40;–0.09); the change-from-baseline mean difference was −0.14 kg/m² (95%CI:-0.23;–0.05). No differences were found between single and multiple health behaviour interventions.

Interventions in the school- and general population setting aiming to reduce only sedentary behaviour and interventions targeting multiple health behaviours can result in significant decreases in sedentary behaviour. Studies need to increase follow-up time to estimate the sustainability of the intervention effects found.

## Introduction

The high prevalence of overweight and obesity among children and adolescents is of worldwide concern [1]. Obese children are more likely than normal-weight children to maintain a high body weight throughout their life making them more vulnerable to health problems in adulthood [2,3].

Studies have demonstrated the relationship between an increase in television viewing or screen time and weight gain [4,5]. Recently, Tremblay *et al*. (2011) suggested that TV viewing of more than 2 hours a day is associated with reduced physical and psychosocial health [6]. Sedentary behaviour may be associated with energy intake, for example through increased snacking during television viewing [4,5]. Also, sedentary behaviour may be associated with energy expenditure by replacing more active pursuits such as playing outside [5,7]. These associations provide rationale for the development of interventions to decrease sedentary behaviour.

Interventions performed in a general setting, for example the school setting, allow for a broad population to be reached, and may contribute to the prevention of overweight and obesity [5,8]. Previous reviews and meta-analyses did not distinguish between interventions that were developed to prevent excessive sedentary behaviour in the general population setting, and interventions that were developed to decrease high levels of sedentary behaviour as part of a treatment for overweight and obese children [6,9-17]. It is important to evaluate interventions specific for the general population setting, to map the preventive effect these interventions may have on sedentary behaviour and overweight prevention. This meta-analysis is the first to provide an overview of the evidence regarding the effects of interventions, implemented in the general population setting, aiming to prevent excessive sedentary behaviour among children and adolescents (0–18 years).

The main study question was: can interventions aiming to prevent high levels of time spent in sedentary behaviours (e.g. television viewing, watching video/DVD), implemented in school- and general population settings, targeting children and adolescents, successfully reduce (1) the amount of sedentary behaviour and (2) Body Mass Index (BMI). Additionally, we explored whether the effects on sedentary behaviour and BMI of single health behaviour interventions (sedentary behaviour only) are similar to the effects of multiple health behaviour interventions (e.g. interventions focusing on sedentary behaviour, dietary intake and physical activity).

## Methods

### Literature search

A literature search was conducted in PubMed, EMBASE, Web of Science, PsycINFO and Cochrane Database of Systematic Reviews in July 2010 using the following key terms: overweight, obesity, intervention, sedentary, television, video, games and children. The complete PubMed search strategy can be found in the additional material [Additional file 1]. The search strategy was adapted for each of the other databases. A search update was performed in March 2011. Included articles and relevant reviews were hand searched for additional eligible studies.

### Inclusion criteria

In order to be included, the study had to be published in a peer-reviewed journal after 31 December 1989. Controlled trials with at least one intervention and one control or non-intervention group were included. The study had to detail an intervention, of any duration, that aimed to reduce the level of sedentary behaviour in children or adolescents (age range 0–18 years). Studies were allowed to also target other behaviours, such as physical activity or dietary behaviours; these studies are referred to as multiple health behaviour studies. These studies needed to explicitly state the intervention elements aimed at sedentary behaviour. Finally, studies had to include a sedentary behaviour outcome (TV viewing, snacks during TV viewing) and/or a weight related outcome (e.g. BMI, BMI-z, percentage overweight children).

Sedentary behaviour included screen time activities and behaviours such as listening to music, reading, ‘sitting around doing nothing’ or talking on the phone. Screen time activities included watching television, DVD/video/HDD viewing, electronic gaming (e.g. game console), computer activities (e.g. internet, gaming) and small screen activities (e.g. PDA, Smartphone).

### Exclusion criteria

Studies performed in laboratory settings, studies with a pre-post test design, studies without a control group, and cohort studies were excluded. We excluded studies aiming at high-risk populations, defined as children or adolescents being overweight or obese, were excluded. Studies comparing normal weight children and overweight or obese children were included when the results for the normal weight children were described separately.

All studies without sedentary behaviour elements in their intervention (e.g. information regarding the influence of advertising or replacement activities for tv-viewing) were excluded. In accordance, studies were excluded when they only targeted physical activity and sedentary behaviour was solely included as an additional outcome.

### Selection process

Titles and abstracts were reviewed independently by two authors (AG and NE) to identify relevant intervention studies. Relevant review articles were identified and reference lists (bibliographies) were screened for additional intervention studies by one author (AG)[18-22]. Authors of relevant design papers were contacted with a request to provide effect papers where available (AG). All studies identified based on the title and abstract were reviewed by both authors (AG and NE) for inclusion and disagreements were discussed with a third party (HR) until consensus was achieved.

### Quality assessment

The Cochrane Collaboration tool for Assessing Risk of Bias was used to assess the quality of the selected studies [23]. For each study seven domains were scored with high, low or unclear risk for bias: sequence generation, allocation concealment, blinding of participants and personnel, blinding of outcome assessment, incomplete outcome data, selective outcome reporting and ‘other’ issues (similarity in baseline characteristics and timing of outcome assessment). These seven domains assess the level of risk regarding selection bias, allocation bias, performance bias, detection bias, attrition bias, reporting bias and other bias. The quality assessment was performed independently by two authors (AG and NE) and the findings were compared and discussed until consensus was achieved.

### Analysis

Study outcomes were quantitatively compared using Review Manager software [24]. Overall mean differences were estimated for the effects of all included interventions. To graph these effect sizes, forest plots were created for study outcomes on (1) sedentary behaviour (minutes per day) and (2) BMI (kg/m²). Separate forest plots were created for (1) post-intervention results of intervention and control group (mean, SD) and (2) post-intervention change-from-baseline difference between intervention and control group (mean, SD). The post-intervention results were used, instead of latest follow-up measurement, for the mean difference estimates to achieve comparable results. Forest plots display the mean and the variance around the mean for each study, and provide a combined estimate with variance of the overall intervention effect.

With regard to sedentary behaviour, 22 studies were included in the post-intervention mean difference estimate and 18 studies were included in the post-intervention change-from-baseline mean difference estimate. For BMI scores, 14 studies were included for the post-intervention mean difference estimate and 14 studies were included for the post-intervention change-from-baseline mean difference estimate. The number of studies included in the above mentioned estimates differed due to study data availability. For example, post-intervention results of a study were given for BMI-z scores only; this study could not be included in the analysis to calculate the overall BMI estimate. Also, a study reporting, for example, post-intervention results only, could not be included in the change-from-baseline analysis.

In addition to the overall mean difference estimate, mean difference estimates for single health behaviour interventions (sedentary behaviour) and multiple health behaviour interventions can be found in each forest plot. These analyses were performed to investigate whether there is a stronger effect of interventions solely focussing on decreasing sedentary behaviour versus interventions combining different health behaviours; previous research has suggested no difference in the effectiveness [25]. Age group (<12 years, 12–18 years) and intervention setting (school setting, home/family setting or combination of settings) were evaluated as potential moderators of the overall intervention effect estimates with post-hoc analyses.

The Cochrane Handbook (version 5.0) was used for guidance regarding missing data and combining data[26]. Available t and p values were used to recalculate missing standard errors. When change-from-baseline data was reported, post-intervention estimates could be calculated. The standard deviation of the baseline estimate was adopted for the follow-up estimate.

Studies with multiple intervention arms were included. The outcomes in each intervention arm were summed and averaged according to the number of participants in each arm. Following guidelines, separately reported results for boys and girls were combined. Child self-report on sedentary behaviour was used whenever both the parent report and child self-report were available. This was done because the majority of the studies used child self-reports.

For sedentary behaviour, some additional calculations were performed. Most studies reported an overall measure for sedentary behaviour. For the few studies that reported on distinct sedentary behaviours, the total post-intervention effects were calculated by summing all distinct estimates. In a similar manner the change-from-baseline result was achieved. To estimate the overall sedentary behaviour change-from-baseline standard error, the coefficient of variation (standard error of the mean divided by the mean) was calculated for every distinct sedentary behaviour change-from-baseline result. When the coefficients of variation of the distinct sedentary behaviour measures differed by less than 0.5, the mean coefficient of variation was estimated and used to calculate the standard error for the overall sedentary behaviour change-from-baseline result. Television viewing was chosen to represent sedentary behaviour when the difference between the coefficients of variation of the distinct sedentary behaviours was larger than 0.5.

If studies only reported one sedentary behaviour (for example TV viewing), this was taken as the sedentary behaviour outcome in the analysis (details on the reported outcomes can be found in [Additional file 2]).

### Heterogeneity

Random effects were estimated, assuming additional variance beyond the set of studies. Estimation of random effects allows for the results to be generalised. The fixed-effect forest plots are available as additional material [Additional file 3, Additional file 4, Additional file 5 and Additional file 6. Heterogeneity statistics are provided in the forest plots (I²) and can be interpreted as low (25 %), moderate (50 %) and high (75 %) variance between studies [27].

## Results

### Search results

**Figure 1:**
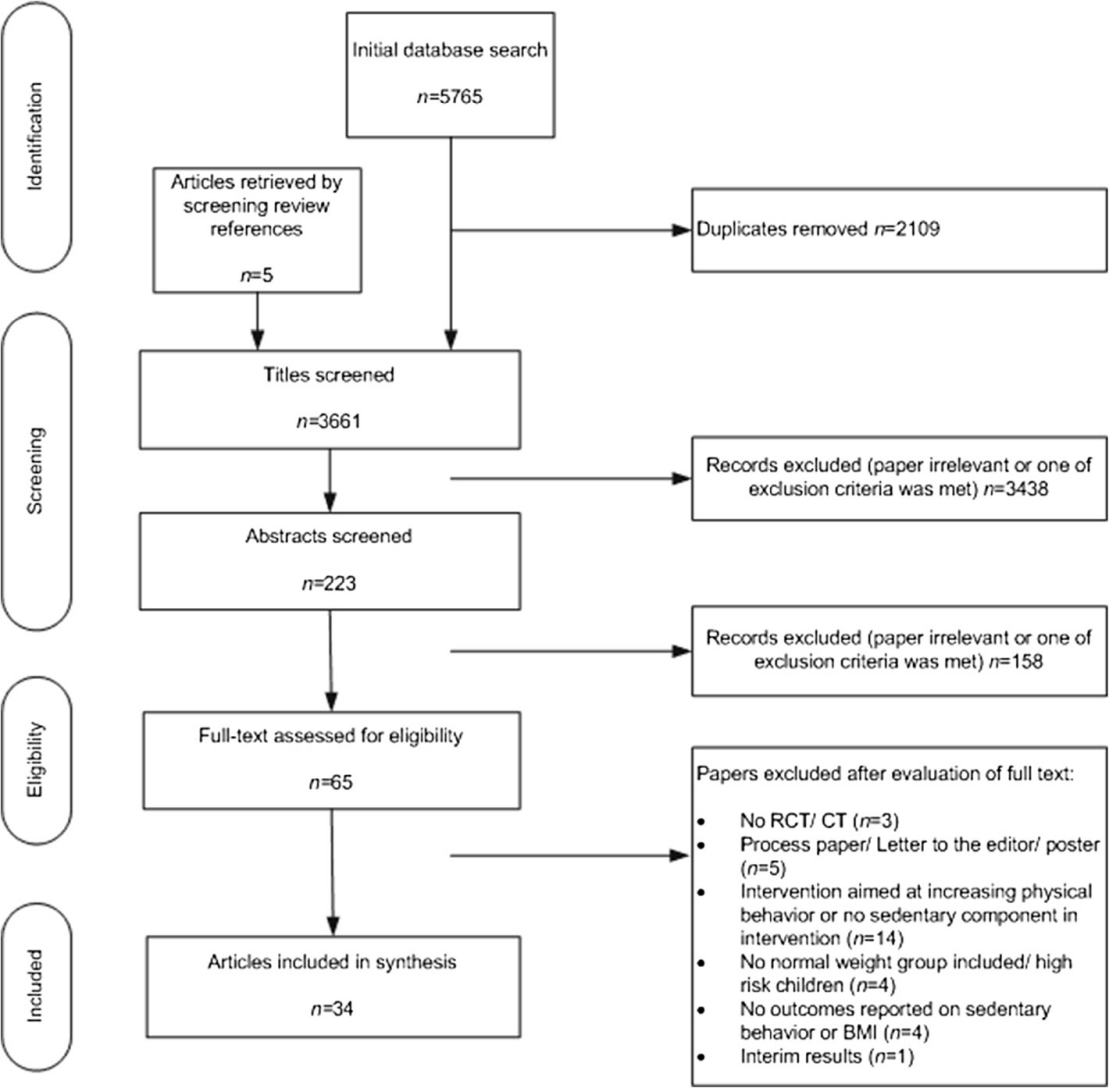
Flow chart of the study selection process.

Figure 1 presents the literature search flowchart using the Preferred Reporting Items for Systematic reviews and Meta-Analyses format [28]. The search revealed 3069 articles. Thirty-four studies reporting 33 different interventions met the inclusion criteria. Four controlled trials [29-32] and 30 randomized controlled trials [33-62] were included. A summary of the general characteristics of each study included can be found in table 1 available in the additional material [Additional file 2.

### Quality assessment

**Figure 2:**
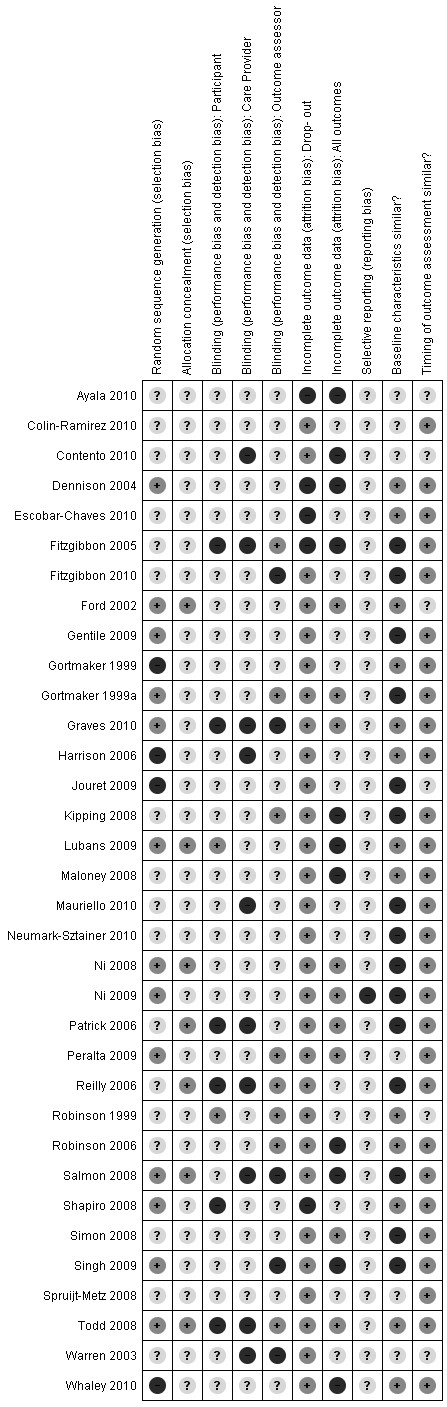
Risk of bias summary.

Figure 2 shows the results of the quality assessment. Sequence generation of the randomisation procedure was adequately reported in 13 studies. Nine studies reported adequate allocation in concealment. However, given the nature of the studies, not reporting allocation concealment does not necessarily mean a study bias. Eight studies reported blinding of the outcome assessor. Dropout rates were reported and rated acceptable in 29 studies. Thirteen studies reported on possible baseline differences between intervention and control groups.

### Sedentary behaviour outcomes

Of the 34 studies, 13 reported a statistically significant effect of the intervention on sedentary behaviour [34,37,38,41,42,48,49,51,53-55,57,58].

**Figure 3:**
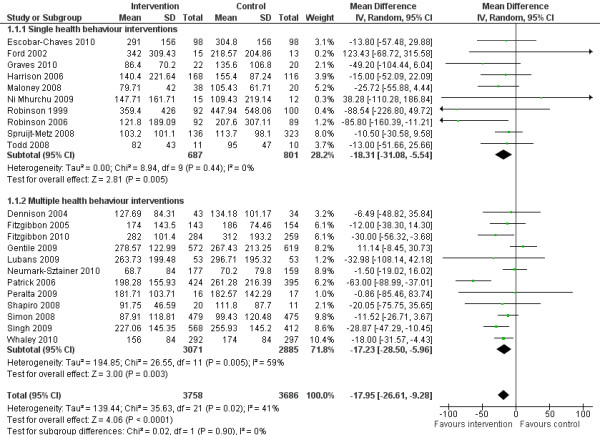
Forest plot, random effects model, comparing intervention and control group on post-intervention sedentary behaviour (minutes per day).

**Figure 4:**
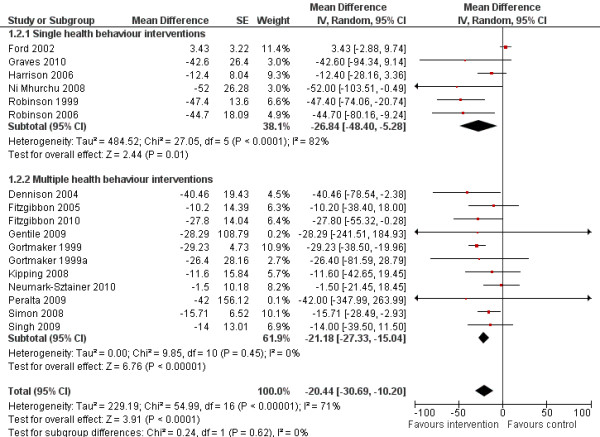
Forest plot, random effects model, comparing intervention and control group on post-intervention change-from-baseline sedentary behaviour (minutes per day).

The random effects model showed a post-intervention mean difference of −17.95 minutes of sedentary behaviour per day in favour of the intervention group (95 % Confidence Interval (CI) = −26.61;–9.28, Figure 3) (standardised mean difference −0.14, 95%CI −0.21;–0.08). Post-intervention change-from-baseline mean difference was −20.44 minutes of sedentary behaviour per day (95%CI = −30.69;–10.20) for the intervention group compared with the control group (Figure 4). There were no significant differences in effects on sedentary behaviour between single and multiple health behaviour interventions. No moderating effects of age or intervention setting were observed for sedentary behaviour (*p* > 0.10).

### *BMI outcomes*

Overall, 6 of the 34 studies reported a significant effect of the intervention on BMI (kg/m²) or BMI-z score [40,44,53,55,57,58].

**Figure 5:**
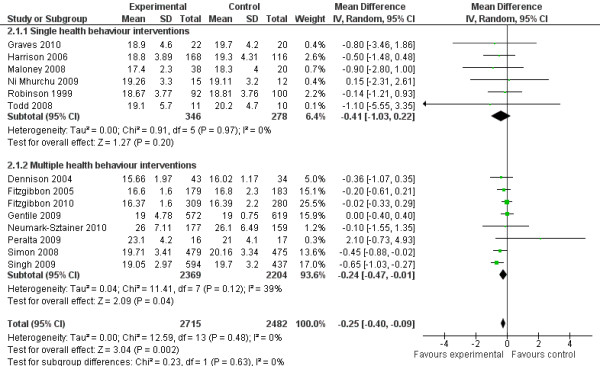
Forest plot, random effects model, comparing intervention group and control group on post-intervention BMI (kg/m²).

**Figure 6:**
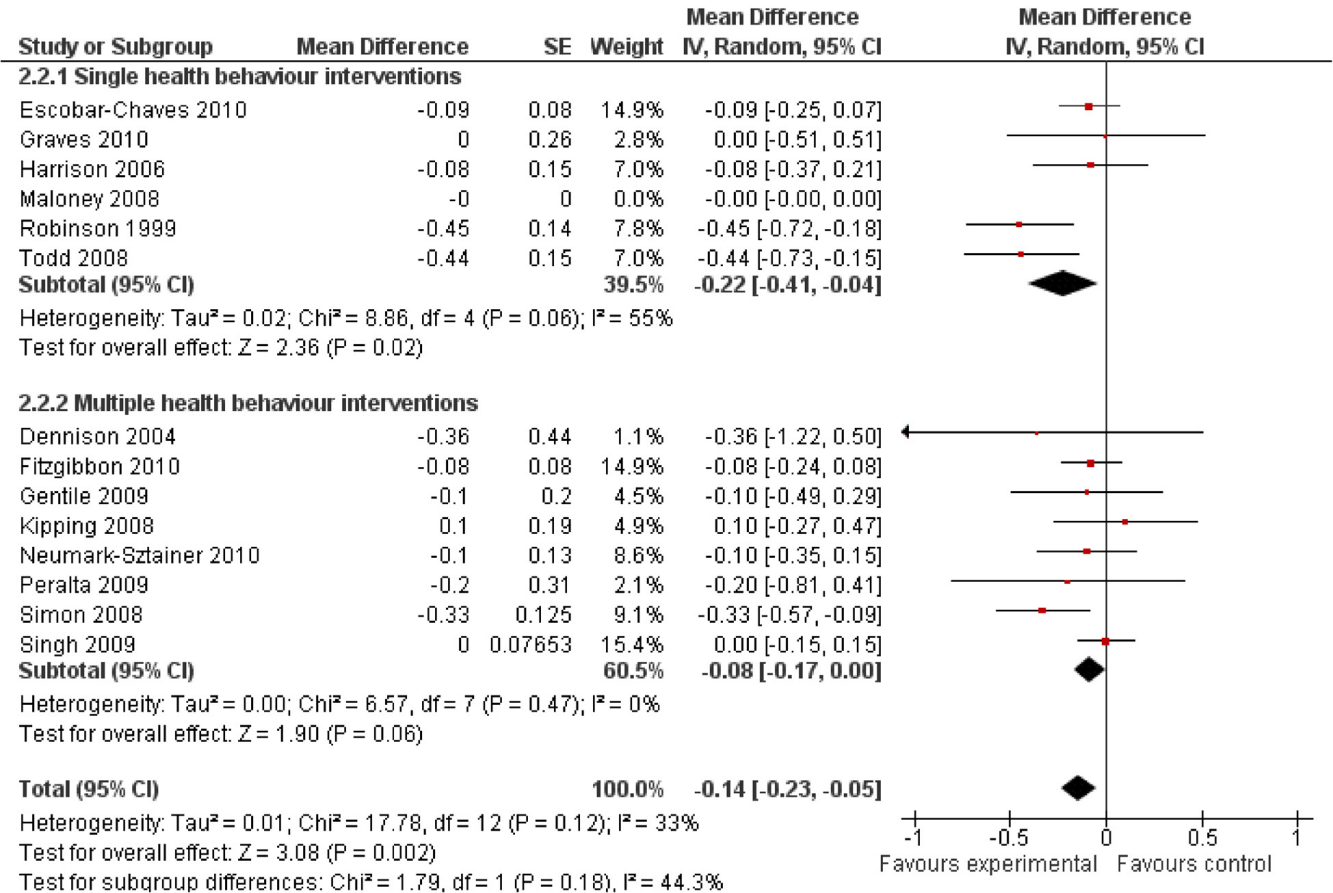
Forest plot, random effects model, comparing intervention and control group on post-intervention change-from-baseline BMI (kg/m²)

The random effects model showed a post-intervention BMI mean difference of −0.25 kg/m² (95%CI = −0.40;–0.09) in favour of the intervention group (Figure 5) (standardised mean difference −0.09, 95%CI −0.14;–0.03). The post-intervention change-from-baseline mean difference was −0.14 kg/m² (95%CI = −0.23;–0.05) in favour of the intervention group (Figure 6). There were no significant differences in effects on BMI between single and multiple health behaviour interventions. No moderating effects of age or intervention setting were observed for BMI (*p* > 0.10).

## Discussion

This meta-analysis is the first to quantitatively compare 34 studies evaluating interventions that were developed to prevent excessive sedentary behaviour (e.g. television viewing, video/DVD) in general population settings among children and adolescents. The results of this meta-analysis show that the interventions aiming to prevent excessive sedentary behaviour may contribute to the prevention of overweight; a significant overall decrease in sedentary behaviour as well as a significant decrease in BMI was found.

Previous reviews and meta-analyses have been published on this topic [6,9-17]. The current meta-analysis is the first to examine the effects of interventions aiming to prevent excessive sedentary behaviour in the general population setting. Sedentary behaviour is increasing across the entire population and prevention of excessive sedentary behaviour is warranted [63,64]. The association between sedentary behaviour and overweight [6], supports the relevance of targeting children and adolescents independent of their current weight status or current amount of TV viewing. Our results showed that efforts, to target the broad child- and adolescent population in decreasing their sedentary behaviour, are successful. The results suggest that in order to improve a healthy weight among children and adolescents, aiming to prevent excessive sedentary behaviour in the general population, e.g. school settings, may be part of an effective approach.

Our study did not show significant differences in the effects on sedentary behaviour or BMI between single health behaviour interventions and multiple health behaviour interventions. This finding is in line with previous research by Krebs *et al.*[25]. But, studies evaluating an intervention specifically designed to prevent high levels of sedentary behaviour could achieve changes in other health behaviours. Effects on other health behaviours were not measured in most studies.

The overall effect estimates of both sedentary behaviour and BMI were not moderated by setting of the intervention and the age of the children targeted with the intervention. The majority of the interventions was aimed at children below 12 years of age and performed in the school setting. In the school setting, sedentary behaviour was generally targeted with individual level interventions such as counselling or tailored feedback. Parents were often involved by means of a newsletter or sometimes by means of more intensive workshops or information meetings. Another approach, although used less frequently, was the home-based intervention. Todd *et al.*[30] found significant results for an intervention targeting both the child’s and the family’s level of sedentary behaviour. These results imply that family members might also benefit from the intervention. Elements from interventions implemented at home included television manager devices. This intervention element can be relatively easily implemented and offered to the parents, and help limit opportunities to be sedentary.

The quality of the studies reporting the interventions varied. The information needed to evaluate risk of bias, was missing in many studies. In almost all studies random allocation of participants was reported, however, the procedures used were not always clearly described. Regarding the blinding of participants, providers and outcome assessors, it should be recognised that the type of research performed within the included studies does not always allow for blinding. Dropout rates were mostly acceptable and well described. Some studies reported being pilot studies and, therefore, reported results on very small samples. This might have added to the differences between groups on baseline characteristics that were reported in some of the studies. For this reason, reporting both unadjusted and adjusted results adds to the quality of the studies.

### Suggestions for future research

Some suggestions for future research should be made. To explore effective intervention elements for the reduction of sedentary behaviour or BMI, studies need to provide details on the intervention and the types of outcome measures taken. A clear description of the intervention should include the health behaviours targeted and the alternatives provided for sedentary behaviour. Owen *et al.*[65] has shown that an effect on health outcomes might already be achieved through replacing sedentary activity with light physical activity. Healy *et al.*[66] found positive effects on health outcomes by increasing the number of breaks during sedentary behaviour. When studies include outcomes measures, such as physical activity levels and dietary intake in addition to sedentary behaviour or BMI, relevant changes in physical activity and other health-related behaviours can be studied in relation to sedentary behaviour.

To improve the understanding of the relationship between sedentary behaviours and weight-related outcomes, mediation analyses of interventions studies can be used. In these analyses the indirect role of sedentary behaviour in the direct relationship between the effect of the intervention and the weight-related outcome is studied.

A drawback of the studies included in this meta-analysis was the short follow-up time in some studies. In our meta-analysis, comparisons were made based on post-intervention results: these post-intervention results were based on measurements taken directly after the intervention or, in some cases, three months after the intervention. The effects found on sedentary behaviour and BMI may alter when longer follow-up results are available for comparison. Based on the studies included in this meta-analysis, providing an estimation of the sustainability of the effects found is therefore difficult.

Our search strategy included terms representing different types of sedentary behaviour, in the included studies the most often targeted behaviour was either TV viewing or screen time (DVD/video viewing and TV viewing). Recently, Salmon *et al.*[14] reported that based on objective measures of activity, only one third of the total sedentary time consists of TV viewing [14]. However, intervention elements to decrease specifically TV viewing may result in more positive effects on health outcomes: increases in TV-time have been significantly associated with several negative health outcomes [5,6].

Considering the weight-related outcome, our study compared outcomes on BMI: BMI-z scores could have provided a more standardized estimate; however, few studies reported these scores.

### Strengths and weaknesses of this meta-analysis

A major strength of this paper is that we could include many studies and were able to estimate an effect based on all interventions combined. Moreover, we reported both adjusted mean differences, taking into account baseline differences, and unadjusted mean differences. In addition, we selected studies performed in school- and general population samples to be able to estimate the effect of interventions aiming to decrease sedentary behaviour for the primary prevention of overweight.

As this meta-analysis is based on published literature, there is a possibility that there is an overrepresentation of effective studies. We did not try to identify unpublished studies. Moreover, the studies included in this meta-analysis reported several distinct types of sedentary behaviour (e.g. computer, television, video). In order to make comparisons, various types of sedentary behaviour were taken together making the effects on unique sedentary behaviours indistinguishable. Therefore, no indication on whether interventions, for example, aiming to reduce computer time are more effective compared to interventions aiming to reduce television-time.

## Conclusion

To summarise, the results indicate that interventions performed in school- and general population settings can help prevent excessive sedentary behaviour and therefore unfavourable health outcomes, among children and adolescents. The intervention can focus on more than one health behaviour and can have a positive effect on sedentary behaviour. Alternatively, interventions can target sedentary behaviour and have relatively small, but positive effects on BMI. Future research should focus on discovering which of the intervention elements, or which combination of elements, is most effective in preventing increases in sedentary behaviour and BMI. Well-designed intervention studies providing details on targeted behaviours and including relevant health-behaviour outcomes with long follow-up are necessary.

This meta-analysis highlights that there are many interventions available to help prevent excessive sedentary behaviour among children and adolescents in a general population setting.

## Abbreviation

BMI, Body Mass Index.

## Competing of interests

The authors declare that they have no competing interests.

## Author contributions

HR and JW originated the idea for the study. AG, WP and NE further developed the study concept and design. AG was responsible for conducting the search, data collection, data extraction, conducting the meta-analysis and drafting the manuscript. AG and NE were responsible for study selection, risk of bias assessment, analysis and interpretation of data. JW contributed to statistical analysis design. HR was responsible for study supervision, overseeing data collection and extraction. All authors contributed to interpretation of the data and critical revision of the manuscript for important intellectual content. All authors have read and approved the final manuscript.

## Supplementary Material

Additional file 1 Search strategy PubMed. Click here for file

Additional file 2 General characteristics of included studies Click here for file

Additional file 3 Forest plot, fixed effect model, comparing intervention and control group on post-intervention sedentary behavior (minutes per day). Click here for file

Additional file 4 Forest plot, fixed effect model, comparing intervention and control group on post-intervention change-frombaseline sedentary behaviour (minutes per day). Click here for file

Additional file 5 Forest plot, fixed effect model, comparing intervention and control group on post-intervention BMI (kg/m²). Click here for file

Additional file 6 Forest plot, fixed effect model, comparing intervention and control group on post-intervention change-frombaseline BMI (kg/m²). Click here for file
