# Supplementary material for: Primary prevention of overweight in children and adolescents: a meta-analysis of the effectiveness of interventions aiming to decrease sedentary behaviour
Source: Int J Behav Nutr Phys Act. 2012 May 28;9:61. doi: 10.1186/1479-5868-9-61 (PMC3462110; doi:10.1186/1479-5868-9-61)
Supplement: Additional file 1 — Search strategy PubMed. [file 1479-5868-9-61-S1.doc]

**Additional file 1** Search strategy PubMed

| Database | Search strategy |
| --- | --- |
| **Pubmed** | (overweight[mesh] OR overweigh*[tw] OR adipos*[tw] OR obes*[tw] OR weight gain*[tw] OR weight chang*[tw] OR weight excess*[tw] OR ((body weight*[tw] OR body mass*[tw] OR bmi[tw])  AND  (toddler*[tw] OR child*[tw] OR infan*[tw] OR pediatr*[tw] OR paediatr*[tw] OR adolesc*[tw])  AND  (prevent*[tw] OR intervent*[tw])  AND  (play and playthings[mesh] OR screen [tw] OR computer*[tw] OR game[tw] OR games[tw] OR gaming*[tw] OR TV[tw] OR television*[tw] OR sedent*[tw] OR sitting[tw] OR reading[tw] OR lazy[tw] OR laying[tw]) |
