# Supplementary material for: Primary prevention of overweight in children and adolescents: a meta-analysis of the effectiveness of interventions aiming to decrease sedentary behaviour
Source: Int J Behav Nutr Phys Act. 2012 May 28;9:61. doi: 10.1186/1479-5868-9-61 (PMC3462110; doi:10.1186/1479-5868-9-61)
Supplement: Additional file 2 — General characteristics of included studies [file 1479-5868-9-61-S2.doc]

**Table 1** General characteristics of included studies

| **Study** | **Methods** | **Participants** | **Intervention** | **Duration intervention/ follow-up** | **Outcome measures of interest** | |
| --- | --- | --- | --- | --- | --- | --- |
|  |  |  |  |  | **Sedentary behaviour¹** | **Anthropometric** |
|  |  |  |  |  |  |  |
| Ayala et al (2010) | RCT  Country: USA | N (control)=227  N (intervention)= IG1 n=200 IG2 n=165 IG3 n=214  Mean age (years): NR (Median 6)  Sex: 51% male  Race: 71% Latino  Lost to follow-up: 48% | Multi-behaviour study/intervention  Intervention group: home-based, family-based & school-based intervention. IG1 MICRO: Each participating family was assigned a *promotera* who visited the home over a 7-month period. A newsletter was reviewed each visit and other material was provided. The parent was guided in setting goals for the next month to improve family lifestyle. The newsletters covered themes such as access and availability to healthy options, increasing FV intake, decreasing TV viewing & increasing active play. Environmental changes included physical changes such as moving a TV out of a child’s bedroom and setting rules and boundaries. After the home visit period, four booster sessions were delivered. Booster calls were made three times. IG2: MACRO; school and community physical environment changes (e.g. child menus in local restaurants). IG3: MICRO + MACRO.  Control group: no intervention. | Duration: 7 months  Follow-up: >12 months post-intervention | TV during dinner¹ | NR |
| Colín- Ramírez (2010) | RCT  Country: Mexico | N (control)=253  N (intervention)=245  Mean age (years): 9.4  Sex: 51.1% male  Race: NR  Lost to follow-up: study based on complete follow-up measurements (n=121 lost to follow-up) | Multi-behaviour study/intervention  Intervention group: school-based & family-based intervention. Individual level: classroom lessons and exercise breaks. Weekly 30-min lessons for 20 weeks. Exercise breaks were 2-10 min. School level: physical education class; regular exercise for 30 min twice a week (moderate to vigorous energy output). Family level: family members to support and reinforce; book with physical exercises to take home. Parents received recommendations for a more active lifestyle, including reducing SB.  Control group: normal curriculum, no intervention. | Duration: 12 months  Follow-up: post-intervention | SST (TV; video; computer)² | NR |
| Contento et al (2010) | RCT  Country: USA | N (control)=574  N (intervention)=562  Mean age (years): 12  Sex: 51% male  Race: 70% Latino  Lost to follow-up: 22.8% | Multi-behaviour study/intervention  Intervention group: school-based intervention. Twenty-four 45-min lessons during 8-10 weeks, some lessons spanned multiple days resulting in about 33 sessions per class. Curriculum addressed selected national science standards in different topics. Activities were performed aimed at taking action. Teachers received a 3-hour pre-intervention session and one session in the middle of the intervention. Teachers were supported by research staff members and received curriculum materials.  Control group: normal curriculum. | Duration: 8-10 weeks  Follow-up: post-intervention | SST (leisure screen time) ² | NR |
| Dennison et al (2004) | RCT  Country: USA | N (control)=34  N (intervention)= 43  Mean age (years): 3.9  Sex: 19% male  Race: NR  Lost to follow-up: 7.4% | Multi-behaviour study/ intervention  Intervention group: parent-based & school-based intervention. 1-hour session each week, 39 weeks with the parents. Half of the session was musical, 10 min. was spent eating a snack and 20 min. was spent participating in an interactive educational session. Seven sessions were on reducing TV viewing. The seven lessons aimed at: turn off of the TV, more family meal time, identifying alternative activities and having discussions.  Control group: safety and injury prevention intervention. | Duration: 7/ 39 weeks  Follow-up: post-intervention | TV^1^/ Video Gaming^1^/ Other¹ | BMI^5^/ BMI-z^b^/ Body Fat^7^ |

**Table 1** *Continued*

| **Study** | **Methods** | **Participants** | **Intervention** | **Duration intervention/ follow-up** | **Outcome measures of interest** | |
| --- | --- | --- | --- | --- | --- | --- |
|  |  |  |  |  | **Sedentary behaviour¹** | **Anthropometric** |
|  |  |  |  |  |  |  |
| Escobar- Chaves et al (2010) | RCT  Country: USA | N (control)=101  N (intervention)=101  Mean age (years): 18.2  Sex: 51.5% male  Race: 43.6% White  Lost to follow-up: 3% | Single-behaviour study/intervention  Intervention group: school-based & family-based intervention. Aims: reduce TV watching, turn of TV when nobody is watching, no TV during meals, no TV in child’s bedroom and to engage in fun non-media related activities. Workshops with e.g.: puppet show, interactive discussion, sharing experiences about family routines, communication skills & alternative activities. At the end [of what?] a fun family plan was made where they [who?] negotiated on child activities and family activities to do as alternative. Newsletters (6 bimonthly).  Control group: no intervention. | Duration: one workshop and 6 bimonthly newsletters  Follow-up: approx. 3 months post-intervention | SST^1^/ TV^1^/ Video^1^/ Video Games^1^/ Computer^1^/ Handheld^1^/ Other^1^ | NR |
| Fitzgibbon et al (2005) | RCT  Country: USA | N (control)=212  N (intervention)=197  Mean age (years): 4.2  Sex: 50% male  Race: 89.9% African American  Lost to follow-up: 26.7% | Multi-behaviour study/intervention  Intervention group: school-based & parent-based intervention. 40-min lesson three times a week. Each week covered a particular topic and included two major components: (1) 20 min. introducing a healthy eating or exercise concept with an activity. In week 10 the topic 'instead of TV…' was discussed. Puppets were used to represent food groups and educate children. (2) 20 min. of ongoing PA. PA included games, e.g. an aerobic trip to the zoo. Parents received weekly newsletters with a homework assignment. Parents received a $5 grocery store coupon for each completed homework assignment. Homework/newsletters mirrored children’s program.  Control group: Weekly curriculum about different health topics for 14 weeks. Topics included seatbelt use and dental care. Parents received a newsletter but did not have to return homework assignments. | Duration: 14 weeks  Follow-up: 12 months, 24 months post-intervention, | TV^1^ | BMI^5^/ BMI-z^b^ |
|  |  |  |  |  |  |  |
| FItzgibbon et al (2010) | RCT  Country: USA | N (control)=323  N (intervention)=346  Mean age (years): 4.3  Sex: 48% male  Race: 94% African American  Lost to follow-up: 7.6% | Multi-behaviour study/intervention  Similar to Fitzgibbon et al (2005); school-based & parent-based intervention. Intervention group: Lessons 2-3 times a week for 14 weeks, curriculum.  Control group: Weekly curriculum about different health topics for 14 weeks. Topics included seatbelt use and dental care. Parents received a newsletter but did not have to return homework assignments. | Duration: 14 weeks  Follow-up: post-intervention | SST^1^/ TV^1^ | BMI^5^/ BMI-z^b^ |
| Ford et al (2002) | RCT  Country: USA | N (control)=13  N (intervention)=15  Mean age (years): 9.5  Sex: 47% male  Race: African American  Lost to follow-up: 10.7% | Single-behaviour study/intervention  Intervention group: primary care & parent-based intervention. A brief 5-10 min. counselling intervention based on discussion of potential problems associated with excessive media use and three brochures were given. 15-20 min. discussion about setting television viewing budgets. And parents received a brochure with steps to reduce child’s TV- watching with a budget. Parents also received an electronic TV time manager to monitor and budget TV time.  Control group: brief 5-10 minutes counselling intervention similar to intervention group. | Duration: 5-10 minutes basic counselling +15-20 minutes extra discussion  Follow-up: 4 weeks post- intervention | SST (TV; video; video games)/ other^1,2^ | NR |

**Table 1 *Continued***

| **Study** | **Methods** | **Participants** | **Intervention** | **Duration intervention/ follow-up** | **Outcome measures of interest** | |
| --- | --- | --- | --- | --- | --- | --- |
|  |  |  |  |  | **Sedentary behaviour** | **Anthropometric** |
|  |  |  |  |  |  |  |
| Gentile et al (2009) | RCT  Country: USA | N (control)=653  N (intervention)=670  Mean age (years): 9.6  Sex: 47% male  Race: 90% White  Lost to follow-up: 25.1% | Multi-behaviour study/intervention  Intervention group: community-based intervention. Several levels: (1) Increasing community awareness and knowledge on preventing childhood obesity. Components included launching project with an event, advertisement campaign, posters, printed materials & public education training/workshops for parents & teachers. (2) School. Teachers received materials and ideas on how to include the core concepts into existing curriculum-> switch was not school based; teachers had to decide themselves what to use. (3) Family. Self-identified goals were rewarded, activities included making a healthy fruit recipe & utilising the screen time box to track time spent in front of screen (TV, video game, computer).  Control group: normal situation. | Duration: 8 months  Follow-up: 6 months post-intervention | SST (TV; video; computer games)^4^ | BMI^5, c^ |
| Gortmaker et al (1999a) (EWKM) | CT  Country: USA | N (control)=289  N (intervention)=190  Mean age (years): 9.2  Sex: 44% male  Race: 91% African American  Lost to follow-up: 15% | Multi-behaviour study/intervention  Intervention group: school-based intervention. Materials were developed to fit in existing curricula. Four behavioural change goals: decreasing intake of foods high in fat, increasing FV intake, reducing TV to <2 hrs/day, increasing moderate and vigorous PA. Lessons on different health subjects. Cards introduced students to new foods and school lunches included these foods. Classroom campaigns were based on reducing TV time, promoting FV and increasing walking (including the family as well). Newspapers based on the lesson content were sent to parents.  Control Group: no intervention, normal curriculum. | Duration: 2 years  Follow-up: post-intervention | TV^2^ | NR |
| Gortmaker et al (1999) | RCT  Country: USA | N (control)=654  N (intervention)=641  Mean age (years): 11.7  Sex: 52% male  Race: 69% White  Lost to follow-up: 18.5% | Multi-behaviour study/intervention  Intervention group: school-based intervention. 16 lessons each year, each lesson had one major subject (specific content unclear in paper). Additionally one lesson was a 2-week ‘reduce TV-watching’ campaign. Classroom lessons were one or two 45-min. periods in duration. Physical Education lesson materials focused on activity and inactivity themes, student self assessment, goal setting and reducing or replacing inactivity. These lessons were thirty 5-min micro-units in duration. Fitness funds were monetary incentives of $400-$500 provided to IG schools in response to teacher submitted proposals.  Control group: normal curriculum. | Duration: 2 years  Follow-up: post-intervention | TV^2^ | BMI^5^/Skinfolds^7^/%Overweight ^d^ |
| Graves et al (2010) | RCT  Country: UK | N (control)=29  N (intervention)=29 | Single-behaviour study/intervention  Intervention group: home-based intervention. Families received a package with instructions to use active video game playing and to encouraging play in a step-powered manner.  Control group: normal video game playing. | Duration: 12 weeks (use of device)  Follow-up: post-intervention | SST (TV; computer; video; games; reading; doing homework)^2^ | BMI^5^/ Body fat^7^ |

**Table 1** *Continued*

| **Study** | **Methods** | **Participants** | **Intervention** | **Duration/ follow-up** | **Outcome measures of interest** | |
| --- | --- | --- | --- | --- | --- | --- |
|  |  |  |  |  | **Sedentary behaviour** | **Anthropometric** |
|  |  |  |  |  |  |  |
| Harrison et al (2006) | CT  Country: Ireland | N (control)=130  N (intervention)=182  Mean age (years): 10.2  Sex: 57% male  Race: NR  Lost to follow-up: 9% | Single-behaviour study/intervention  Intervention group: school-based intervention. Ten 30-min. lessons. Two messages: decrease time spent watching TV and playing computer games and increase PA. Topics: reflecting on spending leisure time and challenging the children to identify realistic alternatives to TV viewing and computer gaming. Self-monitoring, budgeting and goal setting were practiced. Children could receive points. Diaries were part of homework and parents had to sign diary entries. IG schools were visited every two weeks to offer the teachers support. Parents were encouraged in writing to support children.  Control group: continued normal curriculum. | Duration: 16 weeks  Follow-up: post- intervention | SST (TV; video; computer games)^2^ | BMI^5, c^ |
| Jouret et al (2009) | RCT  Country: France | N (control)=410  N (intervention)= IG1 n=750 IG2 n=1030  Mean age (years): 3.8  Sex: 49% male  Race: NR  Lost to follow-up: 30% | Multi-behaviour study/intervention  Intervention group: primary care based intervention. Parents were requested to provide child’s medical records and general practitioner’s information. Children had a medical examination; BMI was determined, parents of at risk children (75-90^th^ BMI percentile) or of overweight children (>90^th^ BMI percentile) were sent a letter to go to the general practitioner (GP). GP provided follow-up care. GP received training in obesity prevention. Reinforcement strategy for parents: Education program, ten 20-min. sessions (5x per year) with learning activities and games about knowledge of food(groups) and health, breakfast, water, sugar, PA & reducing sedentary behaviour. Audio cassette and books reinforced these messages at home. All parents received information on nutrition, PA & obesity in relation to health and well-being.  Control group: no intervention. | Duration: 2 years  Follow-up: post- intervention | NR | % Overweight^a^/ BMI^5^/BMI-z^a^ |
| Kipping et al (2008) | RCT  Country: UK | N (control)=348  N (intervention)=331  Mean age (years): 9.4  Sex: 47.9% male  Race: NR  Lost to follow-up: screen time 52.5% | Multi-behaviour study/ intervention  Intervention group: school-based intervention. Sixteen lessons on healthy eating, increasing PA and reducing TV viewing were taught over 5 months. There were nine lessons on PA, six lessons on nutrition and one lesson on screen viewing. In the PA lessons, children played food-group based games using photos of food. The games reinforced theory from nutrition lessons. The specific TV lesson was about analysing leisure time to identify time spent watching TV and create a list of alternative activities. Teacher materials included lesson plans.  Control group: normal curriculum. | Duration: 16 lessons over 16 weeks.  Follow-up: post-intervention | SST (TV; video; computer games)^2^ | BMI^5^/ % Overweight^a^ |
| Lubans et al (2009) | RCT  Country: Australia | N (control)=66  N (intervention)=58  Mean age (years): 14.1  Sex: 43% male  Race: 94.4% born in Australia  Lost to follow-up: 14.6% | Multi-behaviour study/intervention  Intervention group: school-based intervention with five major components: (1) enhanced school sports program focusing on lifetime physical activities (10 weekly activities e.g. aerobics), (2) information sessions and interactive lecture on PA and healthy diet (at start of each PA session, (3) pedometers, (4) PA and nutrition handbook for participants signed by parents and monthly information for parents & 5) social support. A lecture summarising the 10 messages was given at the end of the program.  Control group: 10-week school sport program. | Duration: 10 weeks  Follow-up: 2 months post- intervention | TV^2^ Electronic games^2^ | NR |

**Table 1** *Continued*

| **Study** | **Methods** | **Participants** | **Intervention** | **Duration/ follow-up** | **Outcome measures of interest** | |
| --- | --- | --- | --- | --- | --- | --- |
|  |  |  |  |  | **Sedentary behaviour** | **Anthropometric** |
|  |  |  |  |  |  |  |
| Maloney et al (2008) | RCT  Country: USA | N (control)=20  N (intervention)=40  Mean age (years): 7.5  Sex: 50% male  Race: 75% White  Lost to follow-up: 10% | Single-behaviour study/intervention  Intervention group: home-based intervention. Equipment for using the Dance Dance Revolution (DDR) was provided to all families and two mats were provided to encourage social and competitive play in the family home. Four sessions were recommended per week for a total of 120 min. per week. Half of the DDR group received five 30-min. one-on-one coaching sessions to see whether coaching encouraged more social and competitive play.  Control group: did not play any DDR (received DDR after intervention). | Duration: 10 weeks  Follow-up: 4.5 months post- intervention | SST (Various activities)^1,2^ | BMI^5^/ BMI-z^9^ |
| Mauriello et al (2010) | RCT  Country: USA | N (control)=1128  N (intervention)=672  Mean age (years): 15.9  Sex: 49% male  Lost to follow-up: 34.3% | Multi-behaviour study/intervention  Intervention group: individual-based intervention. Students answered questions via a 30-minute interactive media-program. Stage-matched tailored feedback was then given on physical activity, fruit & vegetable consumption and TV viewing.  Multi media was used and included animations, audio and video.  Control group: no intervention. | Duration: 2 months  Follow-up: 6 months, 12 months post- intervention | TV^2^ | Height^2^/ weight^2^/BMI^2^/ %Overweight^2,b^ |
| Neumark-Sztainer et al (2010) | RCT  Country: USA | N (control)=174  N (intervention)=182  Mean age (years): 15.8  Sex: 0% male  Lost to follow-up: 6% | Multi-behaviour study/intervention  Intervention group: school-based intervention with eight behavioural objectives throughout the program; more PA; limit SB, increase FV; limit SSB; eat breakfast; decrease portion size and listen to body for signs of hunger; avoid unhealthy weight control behaviours & focus on positive traits. Program consisted of a (16 week) physical education class, individual counselling sessions with motivational interviewing; lunch get together; and minimal parent outreach activities.  Control group: no intervention. | Duration: 9 months  Follow-up: post- intervention | SST^2^/ TV^2^ | BMI^5^/%Overweight^5,b^/ Body fat^7^ |
| Ni Mhurchu et al (2008) | RCT  Country: New Zealand | N (control)=10  N (intervention)=10  Mean age (years): 12  Sex: 60% male  Race: NR  Lost to follow-up: 0% | Single-behaviour study/intervention  Intervention group: home-based intervention. Participants received a package consisting of the EyeToy camera, EyeToy active games and a dance mat. Instruction was to replace usual non-active gaming with active video games (as provided).  Control group: normal situation, no equipment provided. | Duration: 12 weeks  Follow-up: post-intervention | Video^3,1,2^/ video gaming^3, 1, 2^ | % Overweight^5,c^/ BMI^5^/ Waist circumference^5^ |
| Ni Mhurchu (2009) | RCT  Country: New Zealand | N (control)=14  N (intervention)=15  Mean age (years): 10.4  Sex: 62% male  Race: 65% European  Lost to follow-up: 7% | Single-behaviour study/intervention  Intervention group: home-based intervention. Participants were provided with TV time monitors to reduce access to the TV. Tokens were inserted which activated the TV for 30 min. Parents could also block certain programs. Discussion (one session when receiving the device) with parents and researchers on how to use the device, create rules, set TV-free days, record programs to skip adverts & move TV to less accessible location. Recommendation was to monitor TV for <1 hour a day.  Control group: one single session with verbal advice on general strategies to decrease TV watching. | Duration: 1 group meeting & 6 weeks of TV device usage  Follow-up: post intervention (6 weeks) | SST (Various activities)2/ TV^2^ | BMI^5^ |

**Table 1** *Continued*

| **Study** | **Methods** | **Participants** | **Intervention** | **Duration/ follow-up** | **Outcome measures of interest** | |
| --- | --- | --- | --- | --- | --- | --- |
|  |  |  |  |  | **Sedentary behaviour** | **Anthropometric** |
|  |  |  |  |  |  |  |
| Patrick et al (2006) | RCT  Country: USA | N (control)=395  N (intervention)=424  Mean age (years): 12.7  Sex: 48% male  Race: 42% other than white non-Hispanics  Lost to follow-up: 7% | Multi-behaviour study/intervention  Intervention group: individual-based intervention with computer-generated tailored progress plans addressing PA and DI. Two PA, two nutrition and two SB targets were chosen by the computer to compare behaviour to guidelines. According to TTM stage a plan was made, which was signed by the adolescent as a behavioural contract. The next component was delivered by a 16-section printed Teen Guide; mail and telephone using stage-based cognitive and behavioural strategies to support behavioural changes. Monthly reminder phone calls were scheduled during the 12 months: 6 calls were aimed at chosen targets, and remaining calls were on changing plans. Parents were to encourage behaviour through praise, active support and positive role modelling. Adolescents received monetary incentives.Control group: SunSmart program to increase the use of sun protection. | Duration: 12 months  Follow-up: post-intervention | SST (TV; video gaming; sitting talking on the phone; sitting listening to music)^2^ | BMI^5^/ % overweight^b^ |
| Peralta et al (2009) | RCT  Country: Australia | N (control)=17  N (intervention)=16  Mean age (years): 12.5  Sex: 100% male  Race: NR  Lost to follow-up: 0.33% | Multi-behaviour study/intervention  Intervention group: school-based intervention, weekly 60-min. curriculum sessions over 16 weeks and two 20-min. lunchtime PA sessions. PA sessions were organised by 11^th^-grade students to encourage good role modelling. Lessons focused on promoting PA through increasing self esteem and self efficacy, reducing time spent in small screen recreation on weekends, decreasing sugar-sweetened beverage consumption & increasing fruit consumption. Acquisition of self-regulatory behaviours was used throughout the program. Parents received six newsletters with information on program content to motivate them to help their child.  Control group: sixteen 60-min. curricular PA sessions. | Duration: 16 weeks  Follow-up: post-intervention | SST (TV; video; video gaming; computer gaming; internet use; computer use for homework)^2^ | BMI^5^/ Body fat^7^/ Waist Circumference^5^ |
| Reilly et al (2006) | RCT  Country: Scotland | N (control)=277  N (intervention)=268  Mean age (years): 4.2  Sex: 50% male  Race: NR  Lost to follow-up: BMI 8% | Multi-behaviour study/intervention  Intervention group: nursery-based & home-based intervention. Nursery component: enhanced PA program: three 30-min. sessions of PA per week over 24 weeks. Home element: family received a resource pack linking physical play at the nursery and at home. They also received two health education leaflets (one on evidence that PA is low and one on encouraging families to reduce the time spent watching television).  Control group: usual curriculum, no intervention. | Duration: 24 weeks  Follow-up: 6 months post-intervention (only BMI) | SST^3^ | BMI^5^ |
| Robinson et al (1999) | RCT  Country: USA | N (control)=103  N (intervention)=95  Mean age (years): 8.9  Sex: 54.4% male  Race: 70-80% white  Lost to follow-up: 3% | Single-behaviour study/intervention  Intervention group: school-based intervention of eighteen 30-50 min. lessons. Lessons included self-monitoring and self-reporting to reduce time spent playing video games, watching television etc. Additional lessons focused on selective TV viewing. Final lessons were on reducing media use with children as advocates. Each household received an electronic television time manager.  Control group: children received normal curriculum at school and no TV-time manager was provided. | Duration: 2 months  Follow-up: post-intervention | SST^2^/ TV^2^/ Video^2^/ Video gaming^2^/ other^2^ | BMI^5^/ Skinfolds^5^/ Waist circumference^5^ |

**Table 1** *Continued*

| **Study** | **Methods** | **Participants** | **Intervention** | **Duration/ follow-up** | **Outcome measures of interest** | |
| --- | --- | --- | --- | --- | --- | --- |
|  |  |  |  |  | **Sedentary behaviour** | **Anthropometric** |
|  |  |  |  |  |  |  |
| Robinson (2006) | RCT  Country: USA | N (control)=102  N (intervention)=95  Mean age (years): 8.9  Sex: 54% male  Race: 79% White  Lost to follow-up: 2.5% | Single-behaviour study/intervention  Intervention group: school-based intervention of eighteen 30-50 min. lessons. Lessons were divided in four sections: TV awareness, TV turnoff, staying in control & helping others. A weekly 5-10 min. booster session was given in the last four months. Three primary approaches to …..: (1) Decreasing total television and other video time; (2) Selectively decreasing TV time by becoming more selective of context and content and (3) Replacing TV with other activities (no alternatives were given). Non-selective approaches: budgeting, limiting physical access. Selective approaches: certain days or times to watch, restricting to specific content, limiting to particular circumstances. Parents received a newsletter.  Control group: normal curriculum. | Duration: 6 months  Follow-up: post- intervention | TV^1,2^/ Video^1,2^/ Video gaming^1, 2^ | NR |
| Salmon et al (2008) | RCT  Country: Australia | N (control)=62  N (intervention)= IG1 n=66 IG2 n=74 IG3 n=93  Mean age (years): 10.1  Sex: 51% male  Race: NR  Lost to follow-up: BMI data 12% | Multi-behaviour study/intervention  Intervention group: school-based intervention with two intervention-types (both 19 lessons). (1) The BM condition: reduce TV time by 20% (from 2.5->2 hrs/day). Aim of lessons: increase children’s awareness of time issues, learn to self-monitoring time spent in sedentary behaviour and PA, increase awareness of home and community environments in relation to PA and sedentary behaviour choices, improve decision making skills, identify alternatives to sedentary behaviour, increase intelligent watching, identify advertisements on TV & increase advocacy skills. Elements used were: pedometer usage, activity games & poster presentations to younger children. (2) FMS condition: focused on six additional skills (three object control skills and three loco-motor skills). Skills were taught through fun games and maximum involvement for all kids. IG1: BM; IG2: FMS: IG3: BM+FMS.  Control group: no intervention, normal curriculum. | Duration: 9 months  Follow-up: 6 months, 12 months post-intervention | TV^2^/ Electronic games^2^/ Computer use^2^ | BMI^5, a^/% overweight^5, c^ |
| Shapiro et al (2009) | RCT  Country: USA | N (control)=22  N (intervention)= IG1 n=18 IG2 n=18  Mean age (years): 8.7  Sex: 64% male  Race: 32% White  Lost to follow-up: 53% | Multi-behaviour study/intervention  Intervention group: family-based intervention. IG1: Families participated in three weekly educational group sessions (90 min. each). Session 1 introduced the three target behaviours: pedometer usage, estimating beverage sizes and estimating screen time (TV, video game and computer). Session 2 focused on increasing PA and decreasing sedentary behaviour (identifying alternatives, incorporate daily activities). Session 3 focused on the amount of sedentary behaviour and consumption of sugar-sweetened beverages. Each family was given a phone for the study. Instructions were to send 1 SMS a day (with amount on pedometer, number of sedentary behaviours and screen time minutes); an immediate automated SMS feedback message was sent. IG2: same as IG1, but used self-monitoring forms instead of daily SMS and received weekly verbal feedback.  Control group: participated in the 3 sessions but were not expected to self-monitor. | Duration: 8 weeks  Follow-up: post-intervention | SST (Screen time)^1,2^ | BMI(NR)^5,b^ |
|  |  |  |  |  |  |  |

**Table 1** *Continued*

| **Study** | **Methods** | **Participants** | **Intervention** | **Duration/ follow-up** | **Outcome measures of interest** | |
| --- | --- | --- | --- | --- | --- | --- |
|  |  |  |  |  | **Sedentary behaviour** | **Anthropometric** |
|  |  |  |  |  |  |  |
| Simon et al (2008) | RCT  Country: France | N (control)=476  N (intervention)=632  Mean age (years): 11.6  Sex: 49% male  Race: NR  Lost to follow-up: 10% | Multi-behaviour study/intervention  Intervention group: school-based intervention with an educational component focusing on PA and sedentary behaviour. At least two educational classes or debates were devoted to PA. New opportunities for PA during school hours and after school were offered, with a mean of 10 different activities on each site. Sporting events were organised. School transportation by bicycle or by foot was organised (no explicit sedentary behaviour component mentioned).  Control group: normal curriculum. | Duration: 4 years  Follow-up: post-intervention | SST (TV; Video)^2^ | % overweight^5,c^/ BMI^5^/BMI-median^a^/Body fat (FatMassIndex)^7^ |
| Singh et al (2009) | RCT  Country: The Netherlands | N (control)=395  N (intervention)=424  Mean age (years): 12.7  Sex: 50% male  Race:  Lost to follow-up: 19.3% | Multi-behaviour study/intervention  Intervention group: school-based intervention. Individual component: educational program covering 11 lessons on biology and physical education. The first 6 lessons were on increasing awareness and information processing regarding energy balance related behaviours. Students monitored their own behaviour over three days and received feedback. The intervention program guided the students in their choice which of the four behaviours they were going to change and helped formulate implementation intentions. The remaining 5 lessons aimed at facilitating the choice to improve chosen behaviours. To guide the students a computer program was developed. The environmental component was to encourage schools in additional physical education lessons and create changes in and around school cafeterias. Posters suggesting alternative and healthier food choices were delivered to the canteen area and foods were given coloured labels.  Control group: normal curriculum. | Duration: 8 months  Follow-up: 4 months, 12 months post-intervention | SST (TV/ Computer use)^2^ | BMI^5^/ Skinfolds^5^/ Waist Circumference^5^ |
| Spruijt-Metz et al (2008) | RCT  Country: USA | N (control)=323  N (intervention)=136  Mean age (years): 12.5  Sex: 0% male  Race: 72.8% Latino  Lost to follow-up: 7.4% (Whole sample including boys) | Multi-behaviour study/intervention  Intervention group: a school-based and media-based PA intervention was delivered to students during five to seven in-class sessions over five to seven consecutive school days. Students received information about PA and sedentary behaviour and participated in learning activities that supported engagement in PA and reduction of time spent watching TV, sitting in front of computer or 'just sitting around'. Each classroom had to make Public Service Announcement aimed at increasing PA and decreasing physical inactivity in girls just like themselves. Each step in making the Public Service Announcement was aimed to increase both targets of increase in positive meaning in intrinsic motivation. Each lesson had teachable moments delivered in print or verbally (e.g. a fact sheet on health issues).  Control group: normal curriculum. | Duration: 5-7 consecutive days  Follow-up: 3 months post-intervention | SST (TV; Video gaming; Internet)^2^ | BMI-z^5, b^/ BMI Percentile^5^/ BMI(NR)5/ Body fat^7^ |
|  |  |  |  |  |  |  |

**Table 1** *Continued*

| **Study** | **Methods** | **Participants** | **Intervention** | **Duration/ follow-up** | **Outcome measures of interest** | |
| --- | --- | --- | --- | --- | --- | --- |
|  |  |  |  |  | **Sedentary behaviour** | **Anthropometric** |
|  |  |  |  |  |  |  |
| Todd et al (2008) | CT  Country: USA | N (control)=11  N (intervention)=11  Mean age (years): 9.8  Sex: 100% male  Race: NR  Lost to follow-up: 4.5% | Single-behaviour study/intervention  Intervention group: primary care & home-based intervention. A total of five meetings were organized. Meeting one was to provide consent and receive pedometers. Meeting two was to return logbooks and collect BMI. After meeting two, participants were paired. Meeting three was a seminar designed to enhance awareness of electronic media use and to set goals to minimise television use. Awareness education included an interactive 90-min. family session based on TV turnoff network, three follow-up newsletters, ENUFF software (to limit computer and internet use), follow-up call after meeting three, recommendation for progressive reduction in media use to 90 min. per day or less after the first 10 weeks. Participants were contacted by phone each week to encourage and reinforce compliance with the intervention strategy. There was no guidance on PA or DI.Control group: meeting 1, 2 and meeting 4 (measurements), no other information or materials. | Duration: 20 weeks  Follow-up: post-intervention | SST (Electronic media)^2, 4^ | BMI^5^/ Body fat^7^ |
| Warren et al (2003) | RCT  Country: UK | N (control)= 54  N (intervention)= IG1 n=56 IG2 n=54 IG3 n=54  Mean age (years): 6.1  Sex: 51% male  Race: 89% Caucasian  Lost to follow-up: 17% | Multi-behaviour study/intervention  Intervention group: 14-month school-based intervention with 25-min. lessons. IG1: The nutrition group: exploring concepts of health, providing a variety of foods, tasting lessons and games. After that a focus on breakfast and snacking and ending with tooth friendly food. IG2: The physical activity group: insects as theme promoting playground activity, reducing TV viewing, team games, fun PA and quizzes. IG3: Combined groups: both nutrition and physical activity. All intervention groups received a homework book accompanying the lessons. Parents received a newsletter with a resume/an overview of the lessons (each term one newsletter).  Control group: educational program: food in a non-nutrition sense (food in different countries etc). | Duration: 14 months  Follow-up: 1 month post-intervention | NR | % Overweight^5, 9^/ BMI(NR)5/ Skinfolds(NR)^5^ |
| Whaley et al (2010) | CT  Country: USA | N (control)=409  N (intervention)=412  Mean age (years): 1.9  Sex: 50.5% male  Race: 93% of mothers Latino  Lost to follow-up: 28.3% | Multi-behaviour study/intervention  Intervention group: primary care intervention. One-on-one dialogue with WIC staff; six predetermined topics were available for discussion (yummy FV; healthy beverages; less TV; getting up and moving; small, healthy and fun snacks; blank option). At the end of the dialogue a goal and plan for change was developed for the next 6 months. At each meeting a different or the same subject was discussed. Every 6 months the intervention was repeated.  Control group: normal WIC meetings. | Duration: 12 months  Follow-up: post-intervention | TV^2^ | NR |

Abbreviations: RCT, randomized controlled trial; CT, controlled trial; IG, intervention group; CG, control group; BMI, Body Mass Index; SB, sedentary behaviour; SST, sedentary screen time; TV, television;

FV, fruit and vegetables; DI, dietary intake; PA, physical activity; SSB, sugar sweetened beverages, NR, not reported

¶ SST (TV, video, internet); one outcome was reported, in this example overall SST was reported, between brackets the authors definition of SST is given: TV, video and internet. SST/ TV/ Video/ Gaming; all sedentary behaviours mentioned were reported as distinct outcomes.

¹ parent self-report

² child self- report

³ accelerometers

^4^ pedometers

^5^ height/ weight/ waist circumference/ skin folds measured by (trained) research assistants

^a^ use of reference values of a national norm sample

^b^ use of reference values from the CDC charts

^c^ ITOF values (Cole et al, 2000)

^d^ use of percentiles in study data

^7^ Body fat analyser/ DEXA scans

^9^ Unclear
