# Supplementary figures and images for: Primary prevention of overweight in children and adolescents: a meta-analysis of the effectiveness of interventions aiming to decrease sedentary behaviour
Source: Int J Behav Nutr Phys Act. 2012 May 28;9:61. doi: 10.1186/1479-5868-9-61 (PMC3462110; doi:10.1186/1479-5868-9-61)

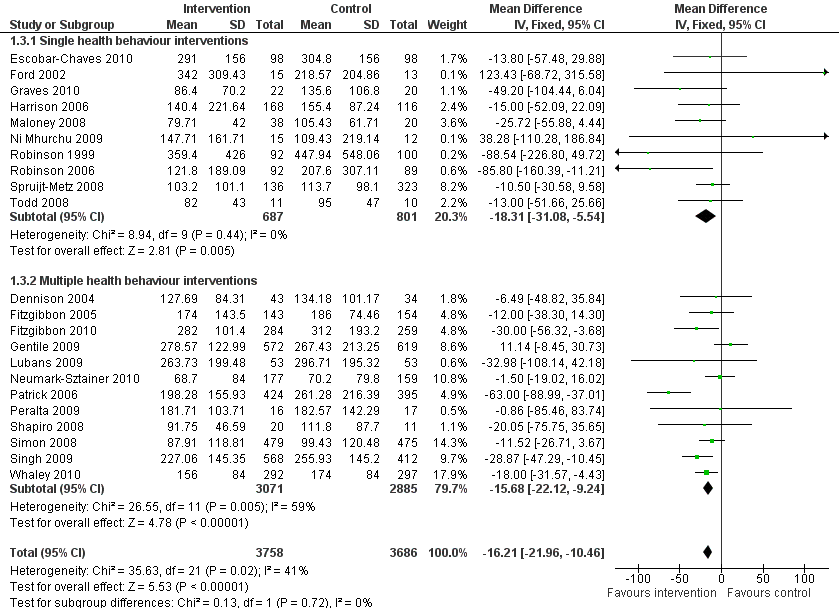

Supplement: Additional file 3 — Forest plot, fixed effect model, comparing intervention and control group on post-intervention sedentary behavior (minutes per day). [file 1479-5868-9-61-S3.png]

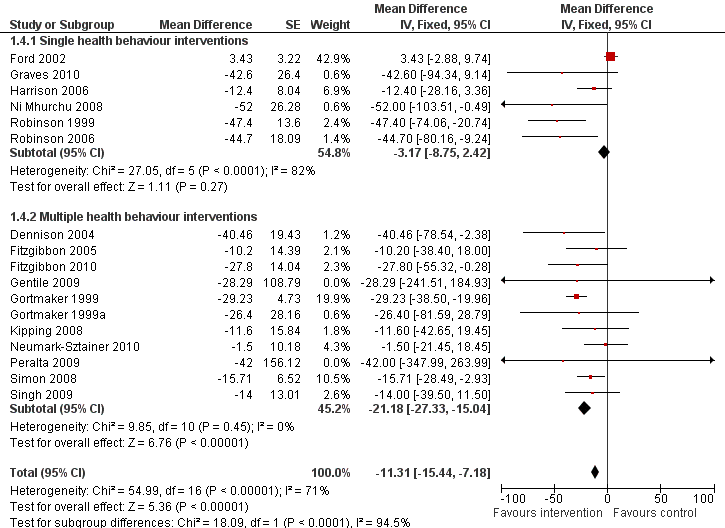

Supplement: Additional file 4 — Forest plot, fixed effect model, comparing intervention and control group on post-intervention change-frombaseline sedentary behaviour (minutes per day). [file 1479-5868-9-61-S4.png]

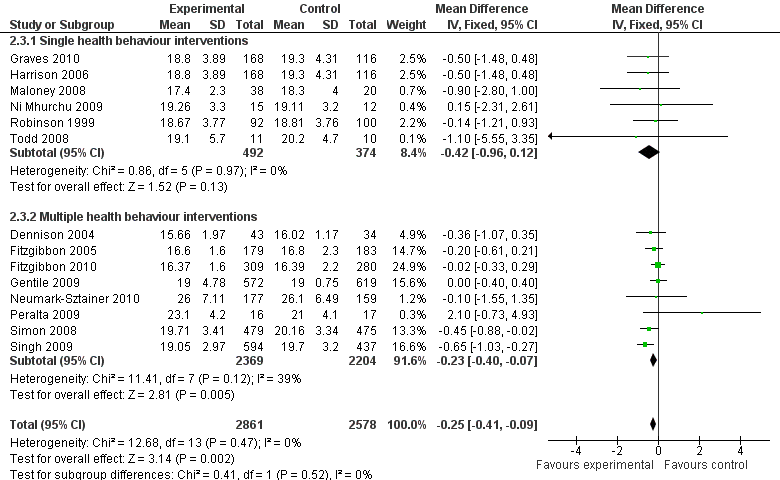

Supplement: Additional file 5 — Forest plot, fixed effect model, comparing intervention and control group on post-intervention BMI (kg/m²). [file 1479-5868-9-61-S5.png]

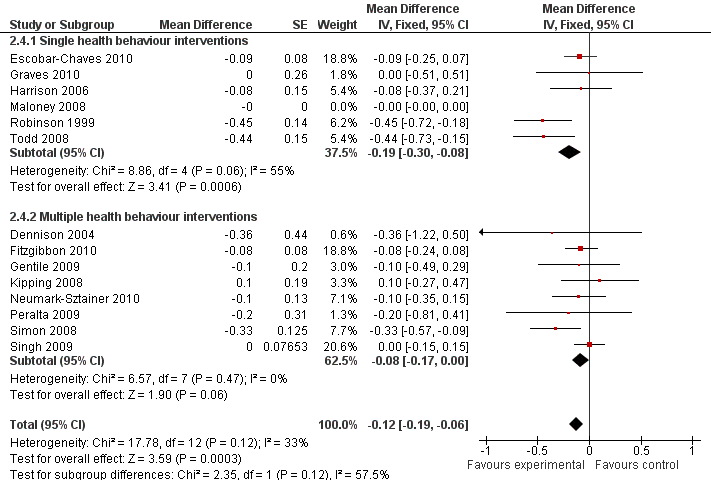

Supplement: Additional file 6 — Forest plot, fixed effect model, comparing intervention and control group on post-intervention change-frombaseline BMI (kg/m²). [file 1479-5868-9-61-S6.png]
